# Efficacy of ablative laser therapy for vulvar lichen sclerosus: a systematic review and meta-analysis of randomized controlled trials

**DOI:** 10.1007/s10103-026-05089-8

**Published:** 2026-11-06

**Authors:** Marie Line Chedid, Laurentia Fidella Averina Setia Santoso, Reina Ibrahim, Baraa Saifi, Heba Sati, Lea Nohra, Sophie Nicolas Moujally, Nancy Emmanuel

**Affiliations:** 1 Medical University of Lodz, Lodz, Poland; 2 Padjadjaran University, Bandung, Indonesia; 3 Frauenklinik, Klinik am Ziegelberg, Aschaffenburg, Germany; 4 Department of Dermatology, Hospital das Clínicas of the Faculdade de Medicina da Universidade de São Paulo, São Paulo, Brazil; 5 Faculty of Medicine, University of Balamand, Tripoli, Lebanon; 6 Faculty of Medical sciences, Lebanese University, Beirut, Lebanon; 7 Universidade Nove de Julho, São Paulo, Brazil

**Keywords:** Vulvar lichen sclerosus, Lichen sclerosus et atrophicus, Laser therapy, Fractional CO2 laser

## Abstract

To evaluate the efficacy of ablative laser therapy, compared to sham laser or topical corticosteroids, or an alternative laser treatment protocol for improving symptom burden and disease severity in women with vulvar lichen sclerosus (VLS). Systematic review and meta-analysis of RCTs. PubMed, Embase, Cochrane Library, Scopus, and Web of Science were searched for RCTs evaluating fractional ablative lasers in adult women with confirmed VLS. Primary outcomes were change in patient-reported symptom burden (visual analog scale [VAS]) and objective disease severity (LS Score, Clinical Scoring System, vulvar-appearance VAS). Mean differences and standardized mean differences were pooled using a random-effects model with restricted maximum-likelihood (REML) estimation of between-study variance and Hartung-Knapp-Sidik-Jonkman (HKSJ) adjustment of confidence intervals; heterogeneity was assessed with I²; risk of bias was assessed with Cochrane RoB 2.0. Three RCTs (151 patients) evaluating fractional CO2 laser versus sham laser, topical clobetasol propionate 0.05%, or low-dose CO2 laser were included, with follow-up of 18 to 32 weeks. Laser therapy produced a non-significant reduction in VAS total (mean difference, 1.04; 95% CI, -4.71 to 6.79; *P*=0.52; I²=58%) and LS severity scores (standardized mean difference, -0.27; 95% CI, -1.47 to 0.94; *P*=0.44; I²=65%). No adverse events were reported. Current evidence from three small, short-duration RCTs does not demonstrate a statistically significant benefit of fractional ablative laser therapy for vulvar lichen sclerosus. Larger, adequately powered trials with standardized outcomes are needed before laser therapy can be recommended as primary or adjunct treatment. PROSPERO CRD420261385742. Not applicable.

**Supplementary Information:**

The online version contains supplementary material available at https://doi.org/10.1007/s10103-026-05089-8.

## Introduction

Vulvar lichen sclerosus is a chronic skin condition affecting the vulva and perianal area in women, especially postmenopausal women. It is characterised by hypopigmented irregular lesions of the skin, often marked by persistent itching, burning, discomfort, and pain. Nonetheless, the exact cause of vulvar lichen sclerosus is unclear; a combination of risk factors contributes to its pathogenesis, such as an overactive immune system, genetic predispositions, hormonal changes, and previous damage or irritation [1].

Over time, the condition often progresses to cause visible skin changes such as atrophy, fissures, and architectural distortion of the vulva. These symptoms often cause serious discomfort and affect everyday life, leading to physical, emotional, and psychological distress [2]. In fact, according to Zivanovic et al., many women reported a decline in self-esteem and sexual well-being due to the appearance and sensitivity of the affected area, leading to the experience of frustration, embarrassment, and withdrawal from social or intimate relationships [3].

The standard treatment is currently the use of topical steroids (propionate 0.05%) to reduce inflammation, help prevent scarring, and further tissue damage [4]. However, the side effects resulting from long term use of steroids may lead to skin thinning or irritation, adding the need for its frequent application, which could lead to poor adherence. Some patients also reported incomplete symptom relief, and relapses are common once the treatment is tapered or discontinued [4].

A potential adjuvant is ablative lasers, such as CO2 and Er: YAG. These lasers work by creating controlled microthermal ablation and coagulation zones within the tissue. This localized thermal injury triggers a wound-healing cascade that promotes neocollagenesis, neoangiogenesis, and epidermal regeneration. In other words, they act by removing affected tissue layers and stimulating dermal remodeling by promoting collagen production and reduction of sclerosus, contributing to restoration of vulvar skin integrity and improving symptoms [2]. Some prior evidence suggests that laser treatment may reduce sclerosis and improve symptoms relative to corticosteroids, with minimal adverse effects [3]. The regulated tissue damage caused by ablative laser treatment poses a theoretical concern regarding disease worsening because lichen sclerosus may show Koebnerization, an isomorphic response, after local trauma. The type of mechanical damage caused by different lasers and the number of sessions may affect the Koebnerization response. It is crucial to assess both treatment-related side effects and efficacy because current clinical research has not determined whether Koebnerization constitutes a clinically significant risk after laser treatment in VLS [5].

However, this evidence is limited: trials of non-ablative laser platforms have shown mixed comparative results [6], and RCT-level evidence specific to ablative lasers remains sparse and conflicting. Nonetheless, the evidence from randomized controlled trials (RCTs) is still modest and conflicting. Previous systematic reviews reported gathered evidences which mostly based on non-randomized trials and case studies; sometimes incorporating randomized trials, which increased the heterogeneity of the study designs [7]. Furthermore, the variation in subjective and objective scorings of the outcomes made the meta-analysis was unable to be performed [8, 9]. Accordingly, this study performs a meta-analysis restricted to randomized clinical trials to assess the effectiveness and safety of ablative laser therapy in treating vulvar lichen sclerosus, with an uniformed subjective and objective scoring analysis.

## Methods

### Search strategy

This systematic review and meta analysis was prospectively registered on PROSPERO. A comprehensive literature search was conducted based on PRISMA 2020 (Preferred Reporting Items for Systematic Reviews and Meta-Analysis) guidelines using the following databases: PubMed, Embase, Cochrane Library, Scopus, and Web of Science in January 2026. For the search strategy the following keywords were used: “vulvar lichen sclerosus” AND “Ablative laser” OR “ Fractional CO2 laser” OR “Er: YAG Laser”. The complete set of search strategies is provided in Supplementary Table 1.

### Inclusion and exclusion criteria

All findings were added into Rayyan and evaluated by two independent reviewers for inclusion. The inclusion criteria were as follows: (1) RCTs articles; (2) studies involving adult women with clinically and/or histologically confirmed vulvar lichen sclerosus; and (3) studies in which the intervention group received fractional ablative laser therapy, either alone or in comparison with another intervention, including sham or standard medical therapy. The exclusion criteria were as follows: (1) Non- RCTs articles; (2) studies lacking appropriate or complete outcome measures; (3) studies that do not report clinical outcomes relevant to treatment effectiveness or safety; (4) studies that were written in foreign languages other than English. After duplicates were removed, both reviewers screened the titles and abstracts to identify eligible studies. Any conflict was resolved by a third reviewer.

### Data extraction

Two reviewers independently extracted data using a standardized form; conflicts were resolved by a third reviewer. The following data were extracted from the included studies: (1) first author and year of publication; (2) country of origin; (3) baseline participant characteristics; (4) total number of participants and group allocation; (5) type of intervention for the experimental and control groups; (6) follow‑up duration; (8) primary and secondary outcomes reported; (9) adverse events and complications. The final extracted dataset was verified by all reviewers, and any discrepancies were resolved after a consensus meeting.

### Outcome definition

The primary outcome was defined as the change in patient-reported symptom burden, measured by the change in the visual analog scale (VAS) total score from baseline to follow-up. VAS is a validated tool commonly used in vulvar lichen sclerosus (VLS) to assess subjective symptom intensity, where patients rate each symptom on a scale from 0 (no symptom) to 10 (worst imaginable symptom) [10]. To allow comparability across studies, we defined the VAS Total as a composite of three symptom domains, typically itching, burning, and pain-related symptoms yielding a standardized total score range of 0 to 30. For each study, change scores were calculated as: ΔVAS = VAS total baseline VAS total follow up.

As studies varied in how symptoms were reported, we applied consistent criteria to derive or standardize VAS Total scores. For all included studies [6, 10, 11], we extracted baseline values and post-treatment scores at a standardized follow-up time point of 12 weeks, except for Burkett et al., for which outcomes were extracted at 24 weeks, the predefined endpoint in that study [10].

In Burkett et al., individual VAS scores were reported for itching, burning, and dyspareunia, each on a 0–10 scale [10]. We computed the VAS Total by summing the means of these three domains, resulting in a 0–30 scale aligned with our primary definition.

Mitchell et al. [11] utilized the Patient Clinical Scoring System (CSS), a validated 0–40 point instrument composed of four symptom domains: pruritus, soreness, burning, and dyspareunia, each rated on a 0–10 VAS. Given its structural and conceptual equivalence to a four-domain VAS composite, we treated the Patient CSS as a valid proxy for VAS Total. To align with the 0–30 scale used in other studies, we proportionally adjusted all means and standard deviations from Mitchell et al. by a factor of 0.75. This conversion assumes proportional equivalence between the four domain patient CSS and the three domain VAS total based on the ration of their maximum possible scores (40 and 30 points respectively), rather than on empirical correlation or a validated cross instrument calibration; no published crosswalk between these instruments currently exists. We treat this as a pragmatic simplifying assumption necessary to enable pooling, not a validated equivalence.

Krause et al. [6] reported a single general VAS score that asked patients, “How much are you disturbed by your symptoms of lichen?” using a 0–10 scale. This was interpreted as a global measure of overall symptom burden and treated as a direct proxy for a composite VAS Total. To ensure consistency with the 0–30 scale used in other studies, we multiplied the reported means by 3 and adjusted the standard deviations accordingly. This transformation similarly assumes that a single global symptom disturbance item is proportionally equivalent to the sum of three separately rated symptom domains, which has not been empirically validated. We treat this, too, as a pragmatic simplifying assumption made necessary by the absence of a directly comparable VAS Total measure in this trial.

For studies that reported baseline and follow-up means with 95% confidence intervals but did not provide standard deviations, standard deviations were derived using the Cochrane-recommended formula: SD = (CI upper − CI lower) / 3.92 × √n [12]. Change scores were then calculated as the difference between baseline and follow-up means. When the standard deviation of the change was not reported, it was estimated using the formula SD_change = √(SD_baseline² + SD_follow-up² − 2r × SD_baseline × SD_follow-up), assuming a correlation coefficient (r) of 0.5, in accordance with Cochrane Handbook guidance [12].

### Secondary outcomes

The secondary outcome was defined as the change in objective clinical severity of vulvar lichen sclerosus, assessed using physician-rated scoring tools. Due to heterogeneity in measurement instruments across studies, we grouped all functionally equivalent severity scales under a unified domain referred to as the “LS Severity Score.” The scoring systems shared the common goal of quantifying observable signs of VLS, including hyperkeratosis, pallor, fissuring, architectural changes, and skin atrophy. To allow for comparability, we treated these tools as conceptually equivalent and analyzed their pooled effect using standardized mean difference (SMD).

Mitchell et al. [11] used the Physician Clinical Scoring System (CSS), a validated 0–12 composite scale comprising three items: hypopigmentation, loss of vulvar architecture, and erosions, each rated on a 0–4 scale. Krause et al. [6] employed the LS Score, also a 0–12 scale, based on four clinical domains: hyperkeratosis, pallor, erosions, and introital narrowing. Burkett et al. assessed clinical severity using a 10-point VAS Appearance score, a subjective physician-rated global impression of vulvar appearance.

For all studies, baseline and post-treatment values were extracted at the final follow-up, which was 12 weeks for Mitchell, Krause, and 24 weeks for Burkett [6, 10, 11].

### Statistical analysis

All statistical analyses were conducted using the R statistical sofware (The Cochrane Collaboration, UK) [12]. Continuous outcomes data were extracted as means and standard deviations (SDs). For outcomes measured on an identical scale (VAS total), mean differences (MDs) with corresponding 95% confidence intervals (CIs) were calculated. For clinical severity, assessed by different scales (e.g., LS Score, Physician CSS or VAS Appearance), standardized mean differences (SMDs) with 95% CIs were calculated. If SDs were not directly reported, they were derived from 95% CIs and sample size using established formulas from the Cochrane Handbook [12]. A random‑effects model was applied to all analyses to account for anticipated clinical and methodological variability across trials in accordance with recommendations from the Cochrane Handbook for Systematic Reviews of Interventions [12]. A p-value of < 0.05 was considered statistically significant.

### Heterogeneity

Statistical heterogeneity was assessed using the *I*^2^ statistic, Cochrane’s *Q* test and Tau-square ( *I*^2^ of 0%, 25%, 50%, and 75% represented no, low, medium and high heterogeneity, respectively).

### Sensitivity and subgroup analysis

A leave-one-out sensitivity analysis was performed by sequentially removing each study and recalculating the pooled estimate and heterogeneity. In this way, we are able to identify which study led to the disproportion contributing to heterogeneity.

### Quality assessment

Risk of bias assessment of the included studies was performed by two independent reviewers using the Cochrane risk-of-bias tool (RoB2), disagreements were resolved by consensus or, if needed, by a third reviewer [13].

### Reporting bias assessment

Funnel plots were not used to assess potential publication bias, as the Cochrane Handbook recommendations suggest that assessment of funnel plot asymmetry is unreliable when fewer than 10 studies are included in a meta-analysis [12].

Clinical trial number is not applicable.

## Results

### Study selection

**Fig. 1:**
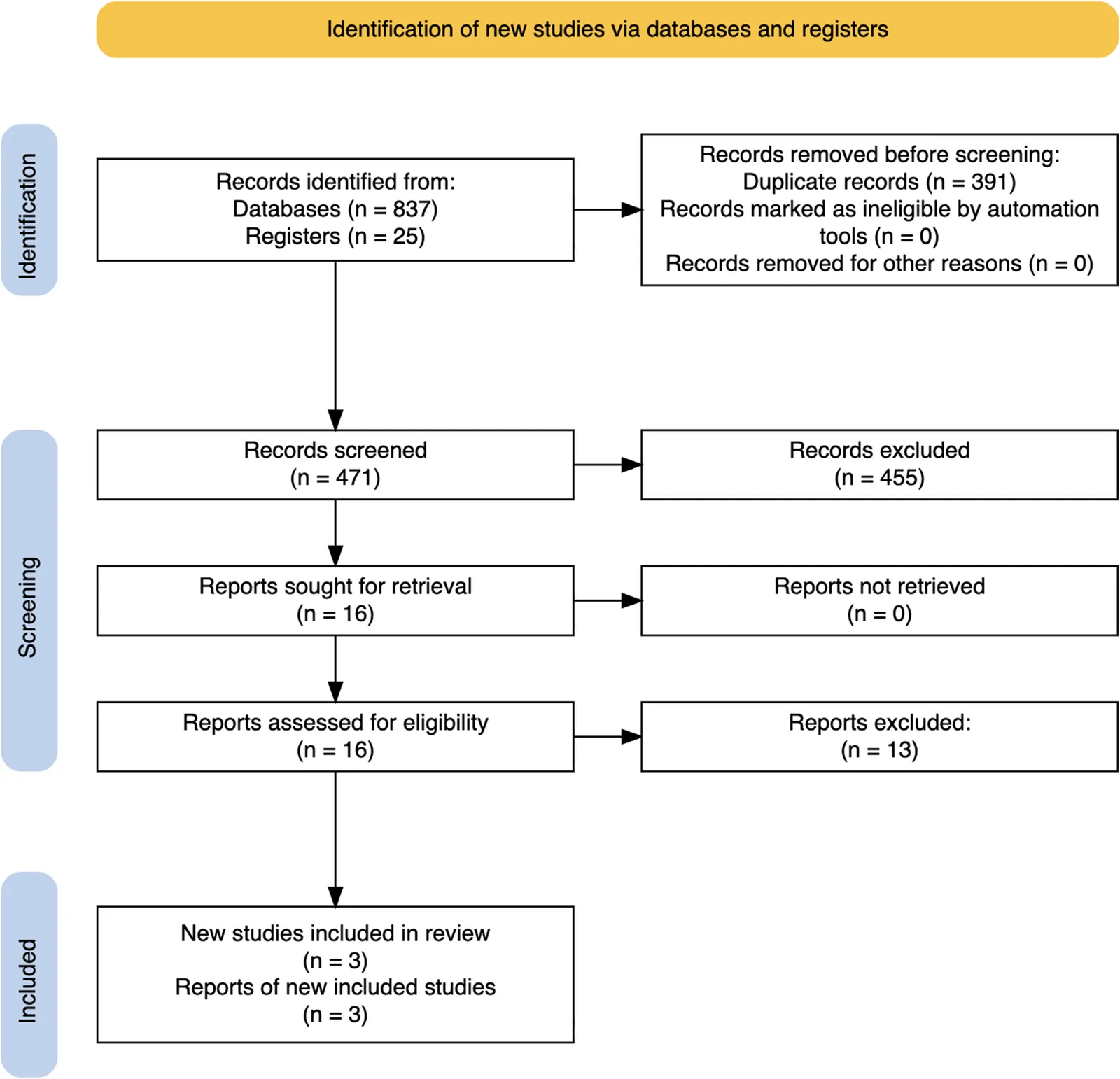
PRISMA flow diagram illustrating the study selection process

The initial search resulted in a total of 862 records. After removing 391 duplicates, 471 articles were screened by two reviewers, and conflicts were resolved by a third. Out of these screened articles, 3 were included based on the inclusion and exclusion criteria mentioned above. Ultimately, 3 studies met the inclusion criteria and were used in this systematic review and meta-analysis. A detailed flow diagram of the study selection process is demonstrated in the PRISMA flow diagram (Fig. 1). The 13 excluded full text reports did not meet one or more of the predefined eligibility criterial outlines in 2.2.

### Baseline characteristics

Three randomized controlled trials (RCTs) assessing a total of 151 patients were included [6, 10, 11]. All studies evaluated the fractional CO₂ laser. Comparators included sham laser, topical clobetasol propionate 0.05% ointment, or low-dose CO₂ laser. Most studies had three laser sessions, with Mitchell et al. being the only one with 5 laser sessions. Follow-up duration ranged from 18 to 32 weeks. The age of participants ranged from 52 to 64.5 years [6, 10, 11]. Table 1 summarizes the baseline characteristics of included studies.

**Table 1:** Summary of included randomized controlled trials on fractional CO2 laser treatment for vulvar lichen sclerosus

Type of Study	RCT	RCT	RCT
Study	Mitchell	Burkett	Krause
Title	Fractionated Carbon Dioxide Laser for the Treatment of Vulvar Lichen Sclerosus	*Clobetasol Compared With Fractionated Carbon Dioxide Laser for Lichen Sclerosus*	*Laser Treatment in Gynaecology – A Randomized Controlled Trial in Women With Symptomatic Lichen Sclerosus*
Total Number of Patients N total (n of intervention / n of control)	ITT: 37 (19 laser / 18 sham) PP: 35 (17 laser / 18 sham)	51 (27 laser / 24 steroid)	63 (34 normal dose / 29 low dose)
Age of Participants (n of intervention/ n of control)	59 years (range: 51–64)	Total: 64.5 ± 10.4 • Laser: 67.6 ± 11 • Steroid: 61.5 ± 8.9	Groups: 52 (range 20–79) and 53 (range 21–80)
Intervention (Type of laser)	Fractionated CO₂ Laser	Fractionated CO₂ Laser	Fractional CO₂ Laser
Laser device	El.En Group laser	SmartXide2–V2-LR	SmartXide Touch C60, MonaLisa Touch, DEKA
Power	Treatment no: 1. 18 watts 2. 20 watts 3. 22 watts 4. 24 watts 5. 26 watts	Treatment no: 1. 26 watts 2. 30 watts 3. 30 watts	24 watts
Pulse duration	Treatment no: 1. 800 microseconds 2. 900 microseconds 3. 1000 microseconds 4. 1000 microseconds 5. 1000 microseconds	Treatment no: 1. 800 microseconds 2. 1000 microseconds 3. 1000 microseconds	400 microseconds
Spacing	Treatment no: 1. 1200 micrometers 2. 1100 micrometers 3. 1000 micrometers 4. 1000 micrometers 5. 1000 micrometers	Treatment no: 1. 800 micrometers 2. 1000 micrometers 3. 1000 micrometers	1000 micrometers
Number of passes	1	1	No information
Treatment area	Vulva region	Visually affected areas of vulvar and perianal lichen sclerosus, except for the glans of the clitoris and clitoral hood, which were spared with at least a 5 mm margin.	Vulva region
Comparator (Corticosteroids or other treatments)	Sham laser treatments: - Power: 4 watts - Duration: 400 microseconds - Spacing: 1500 micrometers	Topical clobetasol propionate (0.05% ointment) over the vulva and perianal area, nightly for 1 month, three times weekly for 2 additional months, then as needed	Low-dose laser (0.5 W; other settings same as intervention group)
Number of laser sessions	5 sessions over 24 weeks	3 sessions, 4–6 weeks apart (2 weeks after biopsy)	3 sessions (three weeks interval)
Follow-up Duration	32 weeks (24-week treatment + 8-week washout)	6 months	18 weeks (with follow-ups at 6 and 12 weeks)
Time since LS diagnosis Median (years)	Median: 8 years (range: 4–16)	Total: 0.56 years (IQR: 3.84) • Laser: 0.8 (4.92) • Steroid: 0.48 (2.84)	Not reported
Primary Outcomes (symptom improvement, lesion seize, healing time…)	Histopathological improvement in biopsy samples (no significant difference between groups) (Δ Score: 20.2; 95% CI 21.14, 1.06, P5.80).	Mean Skindex-29 improvement greater in laser group Effect size: 10.9 points (95% CI: 3.42–18.41, *p* = 0.007)	VAS score (symptom improvement)
Secondary Outcomes	Change in Clinical Scoring System (CSS): no significant improvement in either group Physician CSS (ITT): mean change + 0.70 (95% CI − 0.80 to 2.10; *P* = 0.36) with laser vs. − 0.30 (95% CI − 1.70 to 1.20; *P* = 0.70) with sham; between-group *P* = 0.36	VAS, VSQ, PGI-I, and PGI-S all improved in laser group 89% felt “better/much better” (vs. 62% steroids, *p* = 0.07)81% satisfied (vs 41%, *p* = 0.01) Effect limited to patients with prior steroid exposure	Lichen score, FSFI
Adverse Events/Complications	Only mild, transient discomfort reported	1 laser patient: local burning, treated with steroid1 steroid patient: genital herpes flare-up	31 patients had transient burning during application (resolved < 12 h); no lasting adverse effects
Risk of Bias	- small sample size - short follow-up duration - RCT but lacks placebo comparison	- Selection bias: low (block randomization) - Performance & detection bias: high (unblinded) - Attrition bias: low (1 lost to follow-up) -Measurement bias: moderate (subjective outcomes, unblinded)	Not clearly reported, but likely low; well-defined intervention and comparator, minimal adverse events
Note	- **ITT Analysis**: 3 excluded (missing post-treatment biopsy) - **PP Analysis**: 2 more excluded (missed multiple sessions)	Stratified by prior steroid use; effect on Skindex-29 seen only in previously treated group	Comparison between high- and low-dose laser only; no non-laser control

*ITT intention-to-treat; *PP* per protocol; *VAS* visual analogue scale; *VSQ* vulvovaginal symptoms questionnaire; *PGI-I * patient global impression of improvement; *PGI-S*  patient global impression of satisfaction; *FSFI*  female sexual function index

### Primary outcome: VAS total improvement

**Fig. 2:**
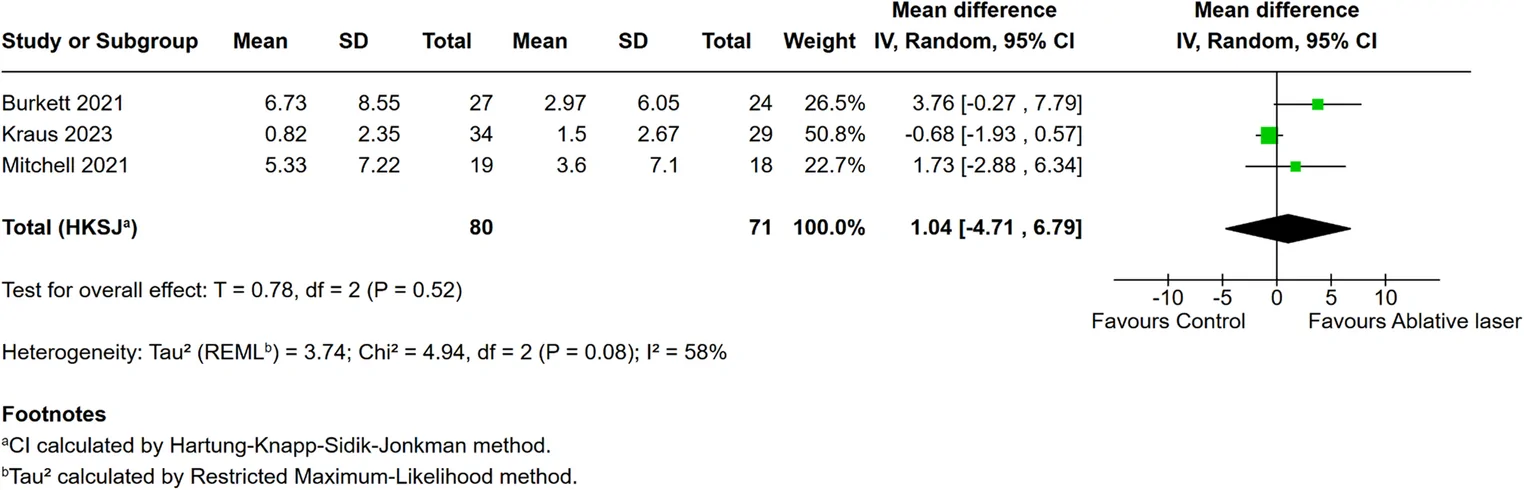
Forest plot comparing mean outcomes between ablative laser and control groups

Only three studies [6, 10, 11] were included in the primary outcome analysis of the VAS total score. Ablative laser therapy was associated with a non-significant improvement in total symptom burden compared to control. The pooled mean difference (MD) was 1.04 (95% CI: −4.71 to 6.79, *p* = 0.52), indicating a trend toward greater symptom improvement in the ablative laser group; however, this difference did not reach statistical significance. Moderate heterogeneity was observed across the included studies, with an I2 of 58%, suggesting some variability in treatment effect estimates (Fig. 2).

### Secondary outcome: LS severity score

**Fig. 3:**
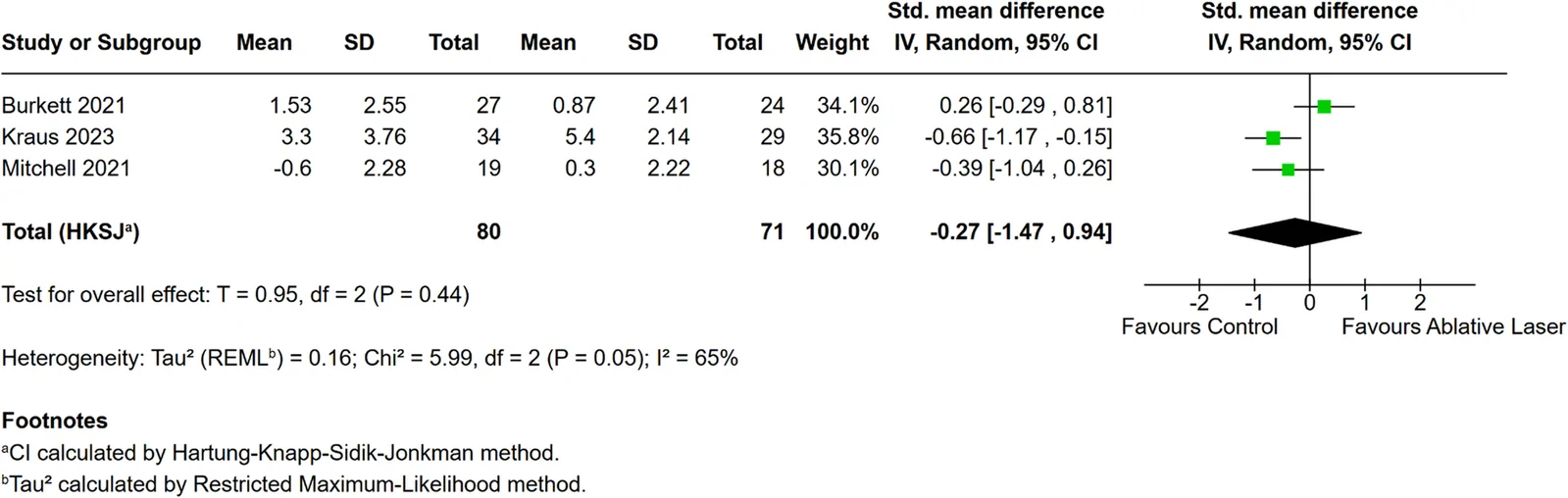
Forest plot of standardized mean differences between ablative laser and control groups

Likewise, the same three studies were analyzed for the change in LS severity score. The pooled standardized mean difference (SMD) was − 0.27 (95% CI: −1.47 to 0.94, *p* = 0.44), indicating no significant difference between ablative laser therapy and control in improving LS severity. Although the pooled effect slightly favored the control group, the difference was not statistically significant. Moderate heterogeneity was observed across the included studies, with an I2 of 65%, suggesting variability in treatment effects among studies [6, 10, 11] (Fig. 3).

### Safety and adverse events

Ablative fractional laser therapy was consistently well tolerated across all three included randomized controlled trials [6, 10, 11], with no serious adverse events or treatment discontinuations reported. The most frequently observed adverse effect was a transient burning sensation during or shortly after the laser procedure, typically lasting up to 12 h and resolving spontaneously without intervention [6, 10, 11]. A single case of localized blistering and burning occurred in the laser arm, which was resolved with topical clobetasol steroid treatment [10]. Notably, one participant in the clobetasol comparator arm of Burkett et al. experienced herpes simplex virus reactivation 1 week post-treatment initiation, in which the steroid treatment was delayed until the episode resolved [10]. No systemic effects, infections, delayed healing, pigmentary changes, or scarring were observed in any study.

### Risk of bias assessment

The risk of bias of the included randomized controlled trials was assessed using the Cochrane Risk of Bias 2.0 (RoB 2) tool [13, 14]. Table 2 summarizes the domain‑level judgments for each study and the risk of bias assessment of the three included studies. All three trials described random sequence generation in detail and were assessed as having a low risk of bias in the randomization process domain [6, 10, 11]. Two studies [6, 10] did not apply blinding of participants or treating clinicians because of the visible cues and procedural differences inherent to laser therapy, and were therefore judged to have some concerns about bias due to deviations from intended interventions. In contrast, Mitchell [11] used a sham laser that reproduced treatment cues to blind participants, but clinicians were not blinded as they had to set the laser parameters; as a result, they earned a low risk rating in this domain. Missing outcome data were minimal across trials. With respect to outcome measurement, all trials used validated instruments, but most did not blind outcome assessors or participants, leading to some concerns for potential detection bias in unblinded settings [6, 10]. For selective reporting, all trials were registered and reported stated outcomes, but none provided a detailed statistical analysis plan, resulting in some concerns for bias in this domain [6, 10]. Overall, Mitchell was assessed as low risk of bias across all domains [11]. Burkett et al. and Krause et al. were each rated as having some concerns, due to open‑label designs, lack of assessor blinding, or incomplete pre‑specification of analysis plans [6, 11]. None of the included trials were judged to be at high risk in any individual domain.

**Table 2:** The domain‑level judgments for each study and the risk of bias assessment of the three included studies

Study	Bias from randomization process	Bias due to deviations from intended interventions	Bias due to missing outcome data	Bias in measurement of the outcomes	Bias in selection of the reported result	Overall risk of bias
Burkett 2021 [10]	Low	Some concerns	Low	Some concerns	Some concerns	Some concerns
Krause 2023 [6]	Low	Some concerns	Low	Some concerns	Some concerns	Some concerns
Mitchell 2021 [11]	Low	Low	Low	Low	Low	Low

### Sensitivity analysis (leave one out analysis)

Sensitivity analysis showed that the pooled result for change in LS severity score remained non-significant after exclusion of individual studies. Notably, removal of Burkett et al. reduced heterogeneity from 65% to 0%, indicating that this study was the main contributor to between-study variability.

#### VAS total

Sensitivity analysis showed that the pooled result for change in VAS total remained non-significant following sequential exclusion of individual studies. Exclusion of Burkett et al., the study comparing laser with clobetasol, reduced heterogeneity from 65% to 0% while maintaining a non-significant pooled effect (MD − 0.51, 95% CI − 8.25 to 7.22; *P* = 0.55). Similarly, exclusion of Krause et al. reduced heterogeneity to 0%, with the effect remaining non-significant (MD 2.88, 95% CI − 9.90 to 15.66; *P* = 0.21). In contrast, exclusion of Mitchell et al. increased heterogeneity to 76%, while the pooled effect remained non-significant (MD 1.11, 95% CI − 26.56 to 28.78; *P* = 0.70). Overall, the findings remained non-significant across all leave-one-out analyses.

#### LS severity

Sensitivity analysis showed that the pooled result for change in LS severity remained non-significant following sequential exclusion of individual studies. Exclusion of Burkett et al., the study comparing laser with clobetasol, reduced heterogeneity to 0%, while the pooled effect remained non-significant (SMD − 0.56, 95% CI − 2.24 to 1.12; *P* = 0.15). Exclusion of Krause et al. resulted in moderate heterogeneity (I² = 55%), with no change in statistical significance (SMD − 0.04, 95% CI − 4.18 to 4.09; *P* = 0.92). In contrast, exclusion of Mitchell et al. increased heterogeneity to 83%, while the pooled effect remained non-significant (SMD − 0.21, 95% CI − 6.09 to 5.67; *P* = 0.73). Overall, the findings remained non-significant across all leave-one-out analyses.

## Discussion

### Summary of findings: trends favoring laser, no statistical significance

In this meta-analysis, only three RCTs were included to analyze the efficacy of ablative laser, mostly fractional CO₂, available for the treatment of VLS. Total trends were in favor of laser vs. control treatments in terms of symptom relief and clinical improvement but did not reach the level of statistical significance in the VAS and MDGlobal categories. All the studies being analyzed had few reports of adverse events, and none of them were severe [6, 10, 11].

### Interpretation of non-significance: possible true clinical modesty of effect disease chronicity and difficulty of treatment, possible issues with patient compliance, limited follow-up durations, moderate heterogeneity between studies limit definite conclusions

Several factors may account for the lack of statistically significant results. First, the actual clinical impact of ablative lasers in VLS may be limited for multiple reasons. While there were some studies that presented positive results, Burkett et al. (2021) found that although satisfaction and symptom relief were higher in patients treated with laser after prior steroid therapy, the overall pooled effect size was small [10]. Second, VLS is chronic and relapsing by definition and also inherently frustrating to treat, with evident but sometimes slow improvement. This intricacy can lead to a lack of observable effects from interventions for short periods of time. Third, adherence by patients to the medications could have affected results, particularly in the comparator arms compared to topical corticosteroids. Non-compliance is notorious in the VLS management as a result of discomfort or fears of chronic steroid use [3].

### Comparison with previous literature

The direction of our pooled point estimates was consistent with several prior individual trial reports of benefit, although unlike some of that literature, our pooled results did ot reach statistical significance.

Burkett et al. (2021) observed that patients in the laser group had a greater symptom relief (89%) as compared to patients treated with steroids (62%) [10]. This contrasts with the sham-controlled trial of Mitchell et al. (2021) that found no significant improvement in either the histology or clinical parameters, which may imply that the improvements reported in other studies could be in part a result of placebo or a hypersensitization of the tissue to the treatment [11].

Zivanovic et al. (2025) similarly evaluated dual Nd: YAG/Er: YAG laser therapy against topical steroid in adult women with lichen sclerosis, stratified by menopausal status. They found that objective clinical improvement (LS score) was similar between premenopausal and postmenopausal women, but postmenopausal women reported significantly greater subjective symptom improvement and treatment satisfaction than premenopausal women [3]. This suggests that psychological and perceptual factors related to age and menopausal status may influence how treatment benefor is subjectively experiences, independent of objective clinical response.

### Clinical implications: ablative laser as potential adjunct or steroid alternative

Given its high safety and efficacy profile and potential to relieve symptoms, ablative laser treatment may complement topical corticosteroids in the treatment of VLS. This approach may be of particular interest in cases with insufficient response to corticosteroids or in patients for whom adherence to long-term topical treatment is difficult. However, the potential benefit of combining laser therapy with corticosteroids has not yet been established. Previous studies have frequently administered topical corticosteroids concurrently with or shortly before fractional CO₂ laser therapy, making it difficult to isolate the independent contribution of laser treatment [11]. Mitchell et al. specifically evaluated fractional CO₂ laser as monotherapy after a treatment washout period. They found no significant benefit over sham treatment in objective disease severity, while acknowledging that a potential role for laser as an adjunct to ultrapotent topical corticosteroids remains to be established [11]. Therefore, whether combined laser–corticosteroid protocols could provide additional clinical benefit or reduce corticosteroid requirements while maintaining disease control remains an important evidence gap. Comparisons with steroid-sparing topical therapies are similarly lacking and should be considered in future studies.

Although there is no evidence that corticosteroids should be replaced by laser therapy as first-line treatment, the potential use of laser as part of a targeted or adjunctive treatment strategy warrants further investigation. Prospective randomized trials with standardized combination protocols, appropriate comparator groups, longer follow-up, and larger populations are needed to better define the role of ablative lasers in the management of VLS.

## Limitations

This meta analysis has several important limitations that should temper the interpretation of its findings. First, the analysis is underpowered, only three randomized controlled trials, comprising a total of 151 patients, met the inclusion criteria. The resulting pooled estimates are accompanied by wide 95% confidence intervals that cross the null for most outcomes, substantially increasing the risk of type ll error and limiting the certainty of any conclusion regarding efficacy. In addition, one of these three trials (krause et al.) compared two active doses of the same laser rather than laser versus an inactive or corticosteroid control, further narrowing the evidence base directly addressing the review’s stated comparison of laser versus sham or standard therapy. Follow up durations were also short, ranging from 6 to 32 weeks, which precludes inference about durability of effect.

Moreover, it is worth noting that moderate heterogeneity was noted across the different studies. The latter was mainly associated with the laser types and variability in follow-up durations (ranging between 6 and 32 weeks). Furthermore, the outcomes measured across studies were not standardized. Indeed, different scoring systems were used in order to assess symptom burden and disease severity (LS Score, Clinical Scoring System [CSS], and vulvar appearance VAS).

Moreover, because Mitchell et al. and Krause et al. did not report a directly comparable VAS Total, proxy scores were derived via proportional rescaling of the Patient CSS and a single global item, respectively. These conversions assume, but do not empirically validate, cross instrument equivalence, and pooled mean difference estimates for the primary outcome should be interpreted with this additional source of uncertainty in mind.

In addition, none of the included trials reported long term clinical outcomes or histologic endpoints. Given that vulvar lichen sclerosus carries a documented risk of malignant transformation to vulvar squamous cell carcinoma, the absence of histologic assessment and of follow-up beyond several months is a substantial evidence gap.

Another limitation which should not be overlooked is the risk of publication bias as most studies originated from single centers. From this perspective, the limitations of this study underline the need for larger, multicenter, well-designed randomized trials. These trials would be able to extend follow-up periods in order to clarify the role of ablative laser therapy in the management of vulvar lichen sclerosus.

## Conclusion

Current evidence from three small, short duration randomized controlled trials suggests that fractional ablative laser therapy may produce modest symptomatic improvement in vulvar lichen sclerosus with an acceptable short term safety profile, but pooled estimates did not reach statistical significance, and no trial assessed long term or histologic outcomes. Until adequately powered, sham controlled trials with standardized protocols and core outcome measures are available, ablative laser therapy should be regarded as a promising but investigational option rather than a substitute for first line topical corticosteroids.

## Supplementary Information

Below is the link to the electronic supplementary material.

Supplementary Material 1

Supplementary Material 2

## Data Availability

No datasets were generated or analysed during the current study.
