# Supplementary material for: Efficacy of ablative laser therapy for vulvar lichen sclerosus: a systematic review and meta-analysis of randomized controlled trials
Source: Lasers Med Sci. 2026 Nov 6;41(1):284. doi: 10.1007/s10103-026-05089-8 (PMC13739930; doi:10.1007/s10103-026-05089-8)
Supplement: Supplementary file 1 [file 10103_2026_5089_MOESM1_ESM.docx]

**Supplementary Table 1:** Search strategies for different databases

| Databases | Search Strategy | Results |
| --- | --- | --- |
| Pubmed | (((((((((((Lichen Sclerosus et Atrophicus[MeSH Terms]) OR (Lichen Sclerosus et Atrophicus)) OR (Lichen Sclerosus)) OR (Lichen Sclerosis)) OR (Lichen Scleroses)) OR (Lichen Sclerosis et Atrophicus)) OR (Vulvar Lichen Sclerosus[MeSH Terms])) OR (Vulvar Lichen Sclerosus)) OR (Kraurosis Vulvae)) OR (Lichen Sclerosus of Vulva)) OR (Vulva Lichen Sclerosus)) AND ((((((((((((((((((((Lasers[MeSH Terms]) OR (Lasers)) OR (Laser)) OR (Pulsed Lasers)) OR (Pulsed Laser)) OR (Continuous Wave Lasers)) OR (Continuous Wave Laser)) OR (Laser Therapy[MeSH Terms])) OR (Laser Therapy)) OR (Laser Therapies)) OR (Pulsed Laser Tissue Ablation)) OR (Laser Photoablation of Tissue)) OR (Laser Tissue Ablation)) OR (Laser Ablation)) OR (ablative laser)) OR (CO2 laser)) OR (fractional laser)) OR (fractional CO2 laser)) OR (Nd:YAG laser)) OR (Er:YAG laser)) | 139 |
| Scopus |  | 351 |
| Web of Science |  | 157 |
| Embase |  | 190 |
| [ClinicalTrials.gov](http://clinicaltrials.gov) |  | 22 |
| WHO ICTRP, |  | 3 |
| ANZCTR |  | 0 |
| grey literature (opengrey.eu, Grey Matters) |  | 0 |
| TOTAL | | 862 |
