# Supplementary material for: Efficacy of ablative laser therapy for vulvar lichen sclerosus: a systematic review and meta-analysis of randomized controlled trials
Source: Lasers Med Sci. 2026 Nov 6;41(1):284. doi: 10.1007/s10103-026-05089-8 (PMC13739930; doi:10.1007/s10103-026-05089-8)
Supplement: Supplementary file 2 [file 10103_2026_5089_MOESM2_ESM.docx]

| **Section and Topic** | **Item #** | **Checklist item** | **Location where item is reported** |
| --- | --- | --- | --- |
| **TITLE** | | |  |
| Title | 1 | Identify the report as a systematic review. | **Title p.1- Title: “Efficacy of ablative laser therapy for vulvar lichen Sclerosus: A systematic review and meta analysis of randomized controlled trials”** |
| **ABSTRACT** | | |  |
| Abstract | 2 | See the PRISMA 2020 for Abstracts checklist. | **Abstract p.2** |
| **INTRODUCTION** | | |  |
| Rationale | 3 | Describe the rationale for the review in the context of existing knowledge. | **Section 1. Introduction (p. 3)** – paragraphs describing LS pathology, limitations of corticosteroids, and laser as adjuvant |
| Objectives | 4 | Provide an explicit statement of the objective(s) or question(s) the review addresses. | **Section 1. Introduction (p. 3)**, last paragraph: "this study performs a meta-analysis of the randomized clinical trials to assess the effectiveness and safety of ablative laser therapy.." |
| **METHODS** | | |  |
| Eligibility criteria | 5 | Specify the inclusion and exclusion criteria for the review and how studies were grouped for the syntheses. | **Section 2.2. Inclusion and Exclusion Criteria (p. 4)** |
| Information sources | 6 | Specify all databases, registers, websites, organisations, reference lists and other sources searched or consulted to identify studies. Specify the date when each source was last searched or consulted. | **Section 2.1. Search Strategy (p. 3–4)** – PubMed, Embase, Cochrane, Scopus, Web of Science; last searched January 2026 |
| Search strategy | 7 | Present the full search strategies for all databases, registers and websites, including any filters and limits used. | **Section 2.1. Search Strategy (p. 4)** + **Supplementary Table 1** (full strategies) |
| Selection process | 8 | Specify the methods used to decide whether a study met the inclusion criteria of the review, including how many reviewers screened each record and each report retrieved, whether they worked independently, and if applicable, details of automation tools used in the process. | **Section 2.2. Inclusion and Exclusion Criteria (p. 4)** – Rayyan, two independent reviewers, conflicts resolved by a third reviewer |
| Data collection process | 9 | Specify the methods used to collect data from reports, including how many reviewers collected data from each report, whether they worked independently, any processes for obtaining or confirming data from study investigators, and if applicable, details of automation tools used in the process. | **Section 2.3. Data Extraction (p. 4)** – two reviewers, standardized form, conflicts resolved by third reviewer, consensus meeting |
| Data items | 10a | List and define all outcomes for which data were sought. Specify whether all results that were compatible with each outcome domain in each study were sought (e.g. for all measures, time points, analyses), and if not, the methods used to decide which results to collect. | **Section 2.4. Outcome Definition (p. 4–5)** and **Section 2.5. Secondary Outcomes (p. 5)** |
|  | 10b | List and define all other variables for which data were sought (e.g. participant and intervention characteristics, funding sources). Describe any assumptions made about any missing or unclear information. | **Section 2.3. Data Extraction (p. 4)** – first author/year, country, baseline characteristics, participants, intervention type, follow-up duration, adverse events |
| Study risk of bias assessment | 11 | Specify the methods used to assess risk of bias in the included studies, including details of the tool(s) used, how many reviewers assessed each study and whether they worked independently, and if applicable, details of automation tools used in the process. | **Section 2.9. Quality Assessment (p. 6–7)** – Cochrane RoB2, two independent reviewers, third reviewer for disagreements |
| Effect measures | 12 | Specify for each outcome the effect measure(s) (e.g. risk ratio, mean difference) used in the synthesis or presentation of results. | **Section 2.6. Statistical Analysis (p. 6)** – MD with 95% CI for VAS Total; SMD with 95% CI for clinical severity |
| Synthesis methods | 13a | Describe the processes used to decide which studies were eligible for each synthesis (e.g. tabulating the study intervention characteristics and comparing against the planned groups for each synthesis (item #5)). | **Section 2.2 (p. 4)** + **Section 2.4–2.5 (p. 4–5)** (grouping by outcome domain) |
|  | 13b | Describe any methods required to prepare the data for presentation or synthesis, such as handling of missing summary statistics, or data conversions. | **Section 2.4. Outcome Definition (p. 4–5)** – scale harmonization (×0.75, ×3 conversions); **Section 2.5 (p. 5)** – SD derivation from CIs, change-score SD estimation per Cochrane Handbook |
|  | 13c | Describe any methods used to tabulate or visually display results of individual studies and syntheses. | **Section 3. Results** with Figure 1 (PRISMA flow), and forest plots/tables in Results |
|  | 13d | Describe any methods used to synthesize results and provide a rationale for the choice(s). If meta-analysis was performed, describe the model(s), method(s) to identify the presence and extent of statistical heterogeneity, and software package(s) used. | **Section 2.6. Statistical Analysis (p. 6)** – Review Manager 5.4, random-effects model, MD/SMD with 95% CI; **Section 2.7. Heterogeneity (p. 6)** – I², Cochrane Q, Tau² |
|  | 13e | Describe any methods used to explore possible causes of heterogeneity among study results (e.g. subgroup analysis, meta-regression). | Section 2.8. Sensitivity and Subgroup Analysis (p. 6) |
|  | 13f | Describe any sensitivity analyses conducted to assess robustness of the synthesized results. | **Section 2.8 (p. 6)** – leave-one-out sensitivity analysis |
| Reporting bias assessment | 14 | Describe any methods used to assess risk of bias due to missing results in a synthesis (arising from reporting biases). | **Section 2.10. Reporting Bias Assessment (p. 7)** – funnel plots not used (per Cochrane Handbook, <10 studies) |
| Certainty assessment | 15 | Describe any methods used to assess certainty (or confidence) in the body of evidence for an outcome. | N/A |
| **RESULTS** | | |  |
| Study selection | 16a | Describe the results of the search and selection process, from the number of records identified in the search to the number of studies included in the review, ideally using a flow diagram. | **Section 3.1. Study Selection (p. 7)** + **Figure 1. PRISMA Flow Diagram (p. 7–8)** |
|  | 16b | Cite studies that might appear to meet the inclusion criteria, but which were excluded, and explain why they were excluded. | N/A |
| Study characteristics | 17 | Cite each included study and present its characteristics. | **Section 3.2. Baseline Characteristics (p. 8)** + **Table 1. Summary of Included Randomized Controlled Trials (p. 8–11)** |
| Risk of bias in studies | 18 | Present assessments of risk of bias for each included study. | **Section 3.6. Risk of bias assessment (p. 12–13)** + **risk-of-bias table (p. 13)** listing Burkett 2021, Krause 2023, Mitchell 2021 |
| Results of individual studies | 19 | For all outcomes, present, for each study: (a) summary statistics for each group (where appropriate) and (b) an effect estimate and its precision (e.g. confidence/credible interval), ideally using structured tables or plots. | **Table 1 (p. 8–11)** for descriptive results; **Forest plots in Sections 3.3 (p. 11) and 3.4 (p. 11–12)** for per-study mean/SD and effect estimates with 95% CIs |
| Results of syntheses | 20a | For each synthesis, briefly summarise the characteristics and risk of bias among contributing studies. | **Section 3.2 (p. 8)** for characteristics + **Section 3.6 (p. 12–13)** for risk of bias of contributing studies |
|  | 20b | Present results of all statistical syntheses conducted. If meta-analysis was done, present for each the summary estimate and its precision (e.g. confidence/credible interval) and measures of statistical heterogeneity. If comparing groups, describe the direction of the effect. | **Section 3.3. Primary Outcome: VAS total improvement (p. 11)** – MD 1.04 (95% CI: −4.71 to 6.79; p = 0.52; I² = 58%); **Section 3.4. Secondary outcome: LS Severity Score (p. 11–12)** – SMD −0.27 (95% CI: −1.47 to 0.94; p = 0.44; I² = 65%) |
|  | 20c | Present results of all investigations of possible causes of heterogeneity among study results. | **Section 3.7. Sensitivity Analysis (Leave one out analysis) (p. 13)** |
|  | 20d | Present results of all sensitivity analyses conducted to assess the robustness of the synthesized results. | **Section 3.7. Sensitivity Analysis (p. 13)** – leave-one-out results showing removal of Burkett reduced I² from 65% to 0% |
| Reporting biases | 21 | Present assessments of risk of bias due to missing results (arising from reporting biases) for each synthesis assessed. | **Section 2.10 (p. 7)** – funnel plots not used (<10 studies per Cochrane Handbook). |
| Certainty of evidence | 22 | Present assessments of certainty (or confidence) in the body of evidence for each outcome assessed. | N/R |
| **DISCUSSION** | | |  |
| Discussion | 23a | Provide a general interpretation of the results in the context of other evidence. | **Section 4.1. Summary of findings (p. 14)** + **Section 4.3. Comparison with previous literature (p. 14–15)** |
|  | 23b | Discuss any limitations of the evidence included in the review. | **Section 4.2. Interpretation of non-significance findings (p. 14)** + **Section 5. Limitations (p. 15)** – small sample, short follow-up, heterogeneity, no histologic outcomes, publication bias |
|  | 23c | Discuss any limitations of the review processes used. | **Section 5. Limitations (p. 15)** – e.g., risk of publication bias from single-center trials; underpowered evidence base |
|  | 23d | Discuss implications of the results for practice, policy, and future research. | **Section 4.4. Clinical implications (p. 15)** + **Section 5. Conclusion (p. 16)** – call for adequately powered, sham-controlled RCTs with standardized protocols |
| **OTHER INFORMATION** | | |  |
| Registration and protocol | 24a | Provide registration information for the review, including register name and registration number, or state that the review was not registered. | The Use of Ablative Laser Therapy in Lichen Sclerosus et Atrophicus of the Vulva: A Systematic Review and Meta-analysis [CRD420261385742] |
|  | 24b | Indicate where the review protocol can be accessed, or state that a protocol was not prepared. | Protocol available via PROSPERO registration |
|  | 24c | Describe and explain any amendments to information provided at registration or in the protocol. | N/R |
| Support | 25 | Describe sources of financial or non-financial support for the review, and the role of the funders or sponsors in the review. | No financial support |
| Competing interests | 26 | Declare any competing interests of review authors. | No competing of interests |
| Availability of data, code and other materials | 27 | Report which of the following are publicly available and where they can be found: template data collection forms; data extracted from included studies; data used for all analyses; analytic code; any other materials used in the review. | The extracted datasets and analytic code used for the meta-analysis are available from the corresponding author upon reasonable request. |

*From:*  Page MJ, McKenzie JE, Bossuyt PM, Boutron I, Hoffmann TC, Mulrow CD, et al. The PRISMA 2020 statement: an updated guideline for reporting systematic reviews. BMJ 2021;372:n71. doi: 10.1136/bmj.n71. This work is licensed under CC BY 4.0. To view a copy of this license, visit <https://creativecommons.org/licenses/by/4.0/>
